# DMAb inoculation of synthetic cross reactive antibodies protects against lethal influenza A and B infections

**DOI:** 10.1038/s41541-017-0020-x

**Published:** 2017-07-06

**Authors:** Sarah T. C. Elliott, Nicole L. Kallewaard, Ebony Benjamin, Leslie Wachter-Rosati, Josephine M. McAuliffe, Ami Patel, Trevor R. F. Smith, Katherine Schultheis, Daniel H. Park, Seleeke Flingai, Megan C. Wise, Janess Mendoza, Stephanie Ramos, Kate E. Broderick, Jian Yan, Laurent M. Humeau, Niranjan Y. Sardesai, Kar Muthumani, Qing Zhu, David B. Weiner

**Affiliations:** 1 Wistar Institute of Anatomy & Biology, Philadelphia, PA USA; 2 Department of Infectious Diseases and Vaccines, MedImmune LLC, Gaithersburg, MD USA; 3 Inovio Pharmaceuticals, Plymouth Meeting, PA USA

## Abstract

Influenza virus remains a significant public health threat despite innovative vaccines and antiviral drugs. A major limitation to current vaccinations and therapies against influenza virus is pathogenic diversity generated by shift and drift. A simple, cost-effective passive immunization strategy via in vivo production of cross-protective antibody molecules may augment existing vaccines and antiviral drugs in seasonal and pandemic outbreaks. We engineered synthetic plasmid DNA to encode two novel and broadly cross-protective monoclonal antibodies targeting influenza A and B. We utilized enhanced in vivo delivery of these plasmid DNA-encoded monoclonal antibody (DMAb) constructs and show that this strategy induces robust levels of functional antibodies directed against influenza A and B viruses in mouse sera. Mice receiving a single inoculation with anti-influenza A DMAb survive lethal Group 1 H1 and Group 2 H3 influenza A challenges, while inoculation with anti-influenza B DMAb yields protection against lethal Victoria and Yamagata lineage influenza B morbidity and mortality. Furthermore, these two DMAbs can be delivered coordinately resulting in exceptionally broad protection against both influenza A and B. We demonstrate this protection is similar to that achieved by conventional protein antibody delivery. DMAbs warrant further investigation as a novel immune therapy platform with distinct advantages for sustained immunoprophylaxis against influenza.

## Nucleic acid delivery: Instant, wide-ranging protection against influenza A and B

A novel innoculation technique involving the injection of antibody-producing plasmid DNA has shown to be effective against influenza in mice. The flu is responsible for up to half a million deaths each year and up to five million cases of severe disease, while also posing a substantial pandemic threat, even with our current repertoire of vaccines. A team of researchers led by Sarah Elliott and David Weiner of The Wistar Institute of Anatomy and Biology, Philadelphia, developed potent plasmid-based constructs that, once injected, entered hosts’ cells and utilized cellular machinery to encode antibodies protective against a range of influenza A and B subtypes. DNA inoculation conferred acute protection from disease, with treated individuals also being immune to subsequent exposure. This approach warrants further investigation as an alternative technology for practical delivery of monoclonal antibody therapeutics.

## Introduction

Influenza virus infection remains a serious threat to global health and the world economy. Annual influenza epidemics result in a large number of hospitalizations, with an estimated 3–5 million cases of severe disease and approximately 250,000–500,000 deaths globally (http://www.who.int/mediacentre/factsheets/fs211/en), with much higher mortality rates possible during pandemics. Despite substantial innovations in treatment and prevention of influenza, licensed antiviral drugs and vaccines do not eliminate the risk of infection. Prompt treatment with therapeutic antiviral neuraminidase (NA) inhibitors can lower influenza morbidity, but these drugs have a limited therapeutic window and are subject to sporadic resistance.^1–3^ Active antiviral immunization with prophylactic influenza vaccines has remarkably lowered seasonal influenza morbidity and mortality at the population level. However, at-risk groups such as infants, the elderly, and otherwise immune-compromised individuals lack optimal adaptive immune responses following vaccination. Furthermore, to counteract the high rate of influenza virus antigenic drift, seasonal vaccines must be re-formulated and re-administered annually at great cost with significant time constraints. Emerging strains of influenza arising from antigenic shift (re-assortment) and cross-species transmission to humans also pose a considerable pandemic threat.^4^ The limited capacity to develop a new vaccine to meet the immediacy and high demand accompanying a pandemic outbreak highlights the global need for new, broad, cost-effective intervention strategies against influenza.^5^

Passive immunization utilizing antibody-based approaches is a notable alternative or adjunct therapy for transient protection against influenza.^6^ Relatively recently, several laboratories have described new classes of influenza-neutralizing monoclonal antibodies that target conserved sites in the hemagglutinin (HA) antigen and cross-react across diverse influenza A or influenza B viruses.^7–10^ Preclinical studies in mice and ferrets reveal these novel cross-reactive antibodies can effectively prevent and treat severe influenza infection, supporting their further study for antibody immunoprophylaxis or immunotherapy in influenza virus-infected humans. While great strides in antibody delivery are being made, the expense of bioprocessed monoclonal antibodies, as well as current requirements for frequent administration, likely pose limitations for universal adoption of this approach and dissemination to global populations. Alternative delivery technologies which co-opt aspects traditionally associated with immunization, such as viral vectored gene therapy, have shown some promise in delivering anti-influenza antibodies in mice,^11, 12^ but permanence concerns and pre-existing anti-vector serology may limit utility of repeatedly using these viral vectors in humans.

A distinct approach to antibody immune therapy which would allow for simplicity of production and lower costs, with high stability and ease of deliverability could be advantageous. In this regard, the technology of DNA-encoded protein antigen delivery has specific advantages as demonstrated by recent successes in the DNA vaccine field: plasmid DNA is well-tolerated and non-integrating, it does not require cold-chain distribution, it can be delivered repeatedly, and it is relatively inexpensive to produce.^13^ However, to date, the ability to produce substantial levels of protein expression systemically from in vivo delivery of plasmid DNA has not been considered feasible.

In this study, we describe construction, development, and in vivo delivery of DNA plasmids encoding optimized influenza-specific broadly neutralizing antibodies, FluA and FluB, that target diverse influenza A and influenza B viruses, respectively. Delivery of FluA DMAb and FluB DMAb results in robust in vivo expression of functional antibodies which protect mice against lethal challenge. We show for the first time that FluA and FluB DMAbs are functionally equivalent to their corresponding protein monoclonal antibodies purified from in vitro cell production. These DMAb impart simultaneous prophylactic cross-protection against diverse strains of influenza A and influenza B virus when given in combination. Furthermore, FluA DMAb and FluB DMAb afford immediate protection without inhibiting host antiviral immunity. The DMAb anti-influenza technology provides a unique alternative strategy to seasonal vaccination, bioprocessed recombinant protein monoclonal antibodies or gene therapy approaches for universal prevention of severe influenza infection. This approach has important implications for protection against and treatment of infectious diseases.

## Results

### FluA and FluB DNA-encoded monoclonal antibodies are expressed in vitro and in vivo

**Fig. 1:**
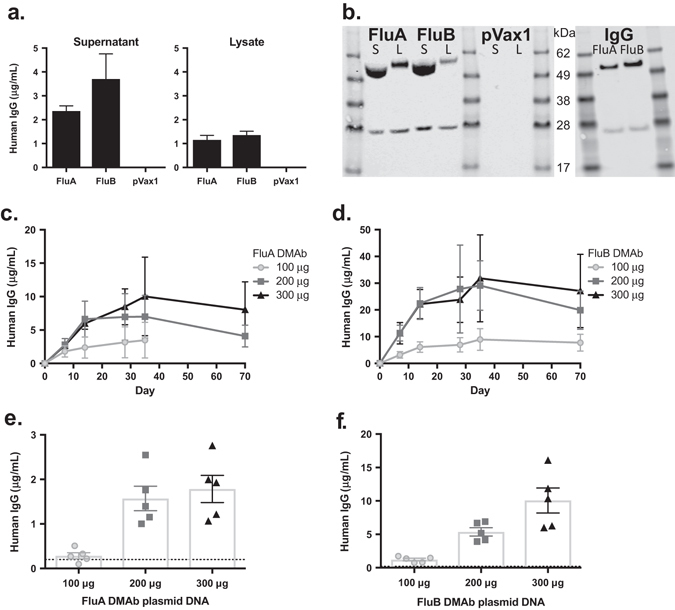
In vitro and in vivo expression of DNA-encoded monoclonal antibody (DMAb) constructs. **a** 293 T cells were transfected with FluA or FluB DMAb plasmid constructs, or empty plasmid (pVax1). Human IgG expression in cell supernatants (*left*) and lysates (*right*) was quantified by ELISA. (*n* = 3 biological replicates, mean ± SEM). **b** Western blot of human IgG heavy-chain and light-chain peptides in reduced DMAb-transfected 293 T cell supernatants (S) and lysates (L) (*left*), and purified protein monoclonal antibody FluA and FluB (IgG, *right*). Samples derive from the same experiment and gels/blots were processed in parallel. **c**, **d** DMAb human IgG in CAnN.Cg-*Foxn1* ^*nu*^/Crl nude mouse sera after intramuscular electroporation (IM-EP) (Day 0) with 100–300 μg of FluA (**c**) or FluB (**d**) DMAb plasmid DNA. Sera were collected up to 35 days post electroporation in mice treated with 100 μg FluA DMAb, and up to 70 days in all other groups. (*n* = 5 animals per group, mean ± SEM). **e**, **f** Levels of DMAb human IgG in BALB/c mouse sera 5 days post administration of 100–300 μg of FluA (**e**) or FluB (**f**) DMAb plasmid DNA. *Dotted line* indicates limit of detection (LOD). (*n* = 5 animals per group, mean ± SEM)

Broadly-neutralizing monoclonal antibodies against influenza A (FluA) and influenza B (FluB) were isolated from human memory B-cells as previously described.^14, 15^ The FluA monoclonal antibody is closely related to a recently published broadly-neutralizing monoclonal antibody, which shows a wide range of HA cross-reactivity due to the binding to the HA stalk and is capable of neutralizing influenza A viruses from both group 1 and group 2 (average IC_50_ of 2.56 μg/mL, data not shown).^10^ The FluB monoclonal antibody was identified and selected based on its ability to potently neutralize influenza B viruses belonging to both Victoria and Yamagata lineages (average IC_50_ of 0.64 μg/mL, data not shown). This antibody binds to a conserved region in the globular head of influenza B HA, and can inhibit viral hemagglutination of red blood cells. To test the utility of DMAb delivery to prevent severe influenza infection, a synthetic DNA transgene encoding either human IgG FluA or FluB was synthesized de novo, and cloned into a mammalian expression plasmid. Multiple modifications were made to enhance DMAb expression including DNA codon optimization, RNA optimization, and formulation of plasmid DNA (Supplemental Fig. S1), as well as plasmid vector design, incorporation of leader sequences for processing and secretion, and CELLECTRA® electroporation techniques.^16, 17^ Quantitative enzyme-linked immunosorbent assay (ELISA) of human IgG in human embryonic kidney 293 T cells transfected with either FluA DMAb or FluB DMAb synthetic constructs confirmed intracellular expression and extracellular secretion of FluA and FluB antibodies, respectively (Fig. 1a). Human IgG Western blot also demonstrated antibody heavy-chain and light-chain expression in transfected 293 T cells (Fig. 1b).

Athymic CAnN.Cg-Foxn1^nu^/Crl nude mice were inoculated with FluA or FluB DMAb plasmid DNA via intramuscular injection at doses from 100 to 300 μg, utilizing intramuscular electroporation (IM-EP) formulated with hyaluronidase to enhance DMAb delivery and expression (Supplemental Figure S1). Peak expression levels in nude mouse sera reached a mean of 10.0 μg/mL (±2.6 SEM) and 31.8 μg/mL (±8.1 SEM) for FluA DMAb and FluB DMAb, respectively. Notably, significant human IgG expression persisted 10 weeks (Fig. 1c, d) and beyond, indicative of the in vivo stability of DNA plasmid and antibody expression.

We next defined the expression of anti-influenza DMAbs in immune-competent BALB/c mice (Fig. 1e, f), an established influenza challenge model. BALB/c mice received 100–300 μg of plasmid DNA via IM-EP. The FluA DMAb construct generated modest levels of human IgG in BALB/c mouse sera as measured 5 days post delivery (300 μg plasmid mean, 1.8 μg/mL ± 0.3 SEM). Similar to what was observed in nude mice, FluB DMAb expression was more robust than FluA DMAb expression 5 days post delivery (200 μg mean, 5.4 μg/mL ± 0.6 SEM; 300 μg mean, 10 μg/mL ± 1.9 SEM). Unlike the stable expression observed in nude mice, serum DMAb levels in BALB/c mice were undetectable 10 days post delivery, likely due to mouse adaptive anti-human-IgG responses against the expressed DMAb. Collectively, these data clearly demonstrated DMAb human IgG was produced at substantial levels in vivo following administration of plasmid constructs.

### In vivo-expressed influenza DMAbs are functionally active and demonstrate broad cross-reactivity

**Fig. 2:**
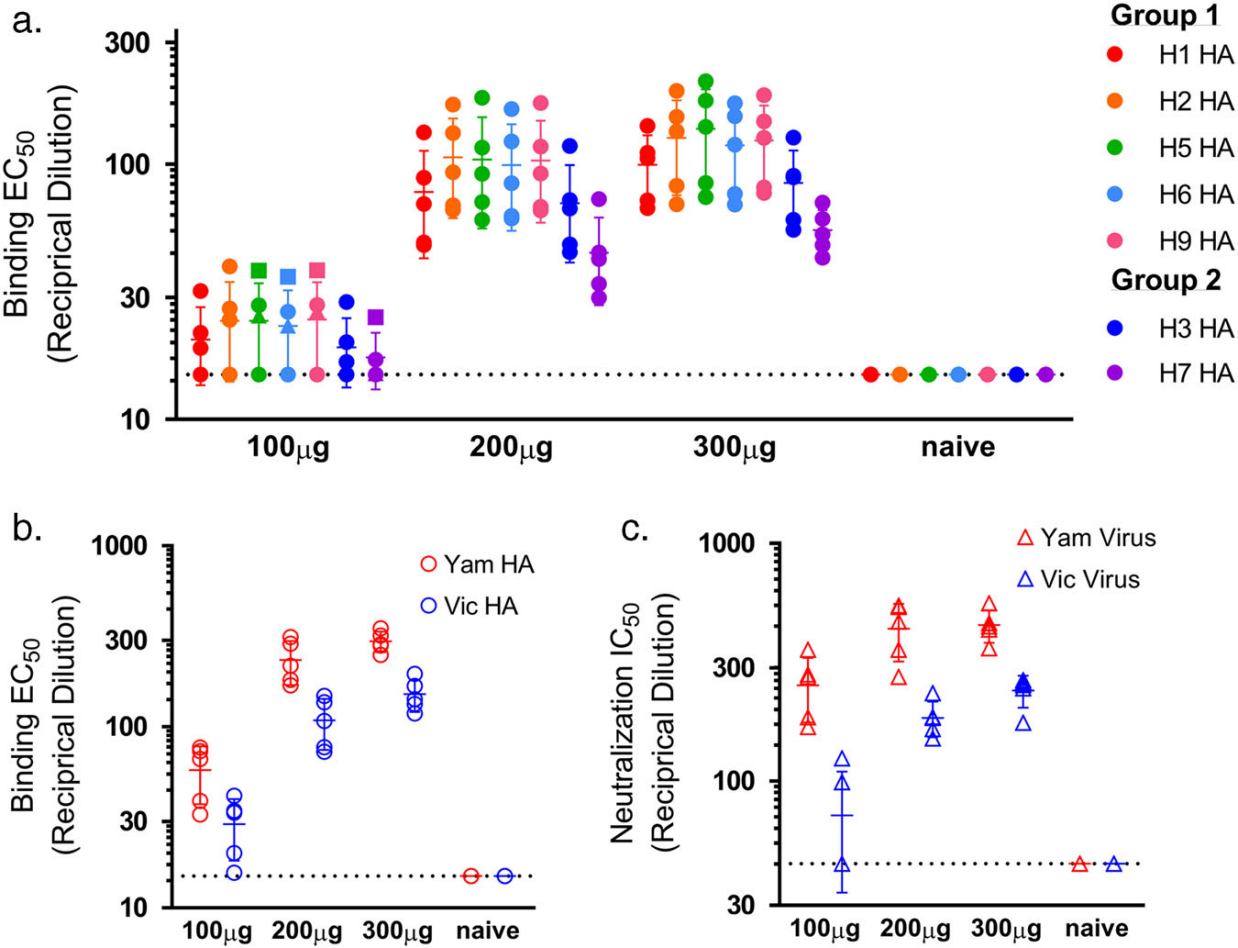
Serum FluA DMAb and FluB DMAb are functional. Functional assays performed with sera from BALB/c mice collected 5 days after treatment with 100–300 μg of FluA DMAb plasmid DNA (**a**) or FluB DMAb plasmid DNA (**b**) and (**c**). **a** ELISA binding EC_50_ values (reciprocal dilution) for individual mouse serum samples to influenza A HA proteins from Group 1 (H1, A/California/07/2009 H1N1; H2, A/Missouri/2006 H2N3; H5, A/Vietnam/1203/2004 H5N1; H6, A/teal/Hong Kong/W312/97 H6N1; H9, A/chicken/Hong Kong/G9/1997 H9N2) and Group 2 (H3, A/Perth/16/2009 H3N2; H7, A/Netherlands/219/2003 H7N7). **b** ELISA Binding EC_50_ values (reciprocal dilution) for individual mouse serum samples to influenza B HA proteins from the Yamagata (Yam B/Florida/4/2006) and Victoria (Vic B/Brisbane/60/2008) lineages. **c** Neutralization IC_50_ values (reciprocal dilution) for individual mouse serum samples against influenza B viruses from the Yamagata (Yam virus B/Florida/4/2006) and Victoria (Vic virus B/Malaysia/2506/2004) lineages. (*n* = 5 animals per group, mean ± SD)

In vitro binding activity in sera collected from DMAb-treated BALB/c mice was determined to test if DMAbs generated in vivo retained cross-reactivity to multiple subtypes of influenza A and both lineages of influenza B. FluA DMAb from sera bound to a comprehensive array of influenza A Group 1 and Group 2 HA antigens from viruses known to infect humans (Table 1) including recombinant trimeric HA from representative seasonal H1, H3 and potentially pandemic H2, H5, H6, H7, H9 influenza isolates (Fig. 2a), as well as recombinant monomeric HA H10 (Supplemental Fig. S2). FluB DMAb in murine sera bound to influenza B HA from representative Victoria and Yamagata lineage viruses (Fig. 2b). Half-maximal effective concentrations (EC_50_) of reciprocal serum dilutions indicated higher binding activity in sera of mice receiving more plasmid DNA, reflecting increased DMAb expression levels.

**Table 1:** Serum DMAbs bind to hemagglutinin antigens from influenza virus subtypes known to cause disease in humans

Hemagglutinin	Zoonotic transmission to humans^a^	Non-human Hosts^b^	DMAb Binding^c^
Influenza A–Group 1			
H1^d^	Sw	Av, Sw	+
H2	Av	Av, Sw	+
H5	Av	Av, Sw	+
H6	Av	Av	+
H9	Av	Av, Sw	+
Influenza A–Group 2			
H3^d^	Sw	Av, Sw, Other	+
H7	Av	Av, Other	+
H10	Av	Av	+
Influenza B			
Victoria^d^	N/A	N/A	+
Yamagata^d^	N/A	N/A	+

^a^ Species from which influenza viruses crossed into humans. Av – Avian, Sw – Swine, N/A – not applicable or unknown

^b^ Non-human species known to carry influenza viruses with the listed hemagglutinin subtype

^c^ DMAb expressed in mouse sera binds to hemagglutinin antigens of the listed subtype by ELISA (+)

^d^ Components of commercially-available seasonal influenza vaccines

The potent in vitro neutralization capabilities of the parent FluB monoclonal antibody allowed for neutralization activity testing whereas the potency of the FluA monoclonal antibody did not allow for differentiation from the non-specific interference of mouse serum in the microneutralization assay. Sera from mice that received FluB DMAb plasmid constructs effectively neutralized both Yamagata and Victoria lineage influenza B viruses in an in vitro cell-based assay (Fig. 2c), with a similar pattern of reactivity as seen in binding assays. After normalizing for human IgG concentration in each sample, the calculated half maximal inhibitory concentration (IC_50_) from mice treated with FluB DMAb plasmid (0.015 μg/mL for B/Florida/4/2006 and 0.030 μg/mL for B/Malaysia/2506/2004) was similar to that of purified recombinant FluB monoclonal antibody (0.011 μg/mL for B/Florida/4/2006 and 0.047 μg/mL for B/Malaysia/2506/2004). The HA-binding activity and neutralization titers of human IgG observed in serum of mice receiving FluA or FluB DMAb plasmid constructs confirmed in vivo expression of functional DMAb and demonstrated the remarkable, broad cross-reactivity of these novel anti-influenza FluA and FluB antibodies.

### Influenza DMAbs protect mice from diverse influenza A and influenza B lethal challenges

**Fig. 3:**
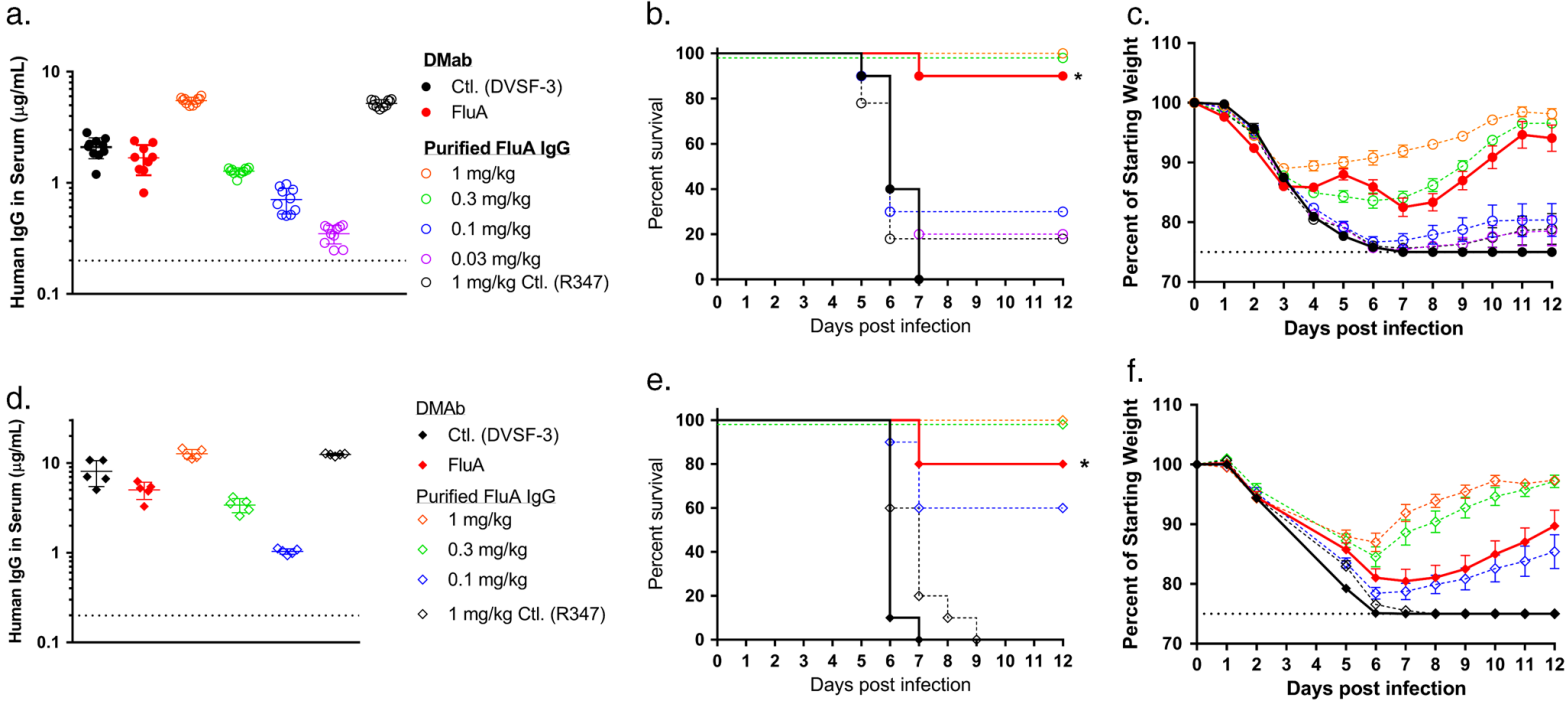
FluA DMAb protects mice from diverse lethal influenza A challenges. BALB/c mice were treated with FluA DMAb plasmid DNA (*closed symbols*) 4–5 days prior to intranasal infection with A/California/7/2009 H1N1 (**a–c**) or re-assorted rA/HongKong/8/68 × PR8 H3N1 (**d–f**). 1 day prior to infection, separate mice received 0.03–1 mg/kg FluA protein monoclonal antibody i.p. (*open symbols*). Mice treated with 300 μg irrelevant DMAb (DVSF-3) or 1 mg/kg non-specific protein monoclonal antibody (R347) served as controls. **a**, **d** Human IgG in mouse sera at the time of influenza infection. *Dotted line* indicates LOD. (**a** *n* = 10 animals, **d** *n* = 5 animals per group, mean ± SD). **b**, **e** Kaplan–Meier survival curves of BALB/c mice challenged with influenza A (*n* = 10 animals per group, *p ≤ 0.0001 FluA DMAb versus Control DMAb). **c**, **f** Weight of BALB/c mice following influenza A challenge. *Dotted line* indicates 25% maximum weight loss. (*n* = 10 animals per group, mean ± SEM)

To assess the in vivo utility of the technology, DMAb-treated animals were evaluated in lethal influenza challenge models. Animals were administered 300 μg FluA DMAb plasmid DNA or an irrelevant DMAb construct against dengue virus (DVSF-3)^17^ via IM-EP, then challenged with a lethal dose of A/California/7/2009 H1N1 (A/CA/09 H1) 4 days post administration (Fig. 3). For direct in vivo comparison of DMAb and recombinant IgG, a dilution series of FluA protein monoclonal antibody was delivered intraperitoneally (i.p.) to separate groups of mice 1 day prior to infection. Serum samples obtained from all animals at the time of infection showed that FluA DMAb construct delivery resulted in similar mean human IgG concentrations and HA binding activity as observed in mice treated with 0.3 mg/kg of recombinant FluA IgG (Fig. 3a, Supplemental Fig. S3). When challenged with a lethal dose of A/CA/09 H1 virus, FluA DMAb treatment provided a 90% survival benefit whereas all animals treated with control DVSF-3 DMAb succumbed to infection (Fig. 3b). Corresponding to human IgG expression levels, the FluA DMAb treatment and 0.3 mg/kg of FluA purified protein exhibited similar protection from lethality and influenza-induced weight loss (Fig. 3c).

Expanding these results with another clinically relevant influenza A virus, a similar study was performed using a lethal challenge of rA/Hong Kong/8/68 H3N1 (rA/HK/68 H3) 5 days post DMAb administration. Again at the time of infection, human antibody levels showed FluA DMAb and 0.3 mg/kg of recombinant FluA IgG at similar concentrations in sera (Fig. 3d). After lethal rA/HK/68 H3 challenge, FluA DMAb provided a significant survival benefit compared to DMAb control (80% survival rate with FluA DMAb versus 0% survival rate with DVSF-3 DMAb) (Fig. 3e). These results show FluA DMAb effectively protected animals from lethal infection by two clinically relevant seasonal H1 and H3 subtypes known to cause disease in humans, and importantly demonstrates similar in vivo antiviral activity of FluA antibody generated via the DMAb platform versus bioproecessed recombinant FluA antibody-delivered i.p.

**Fig. 4:**
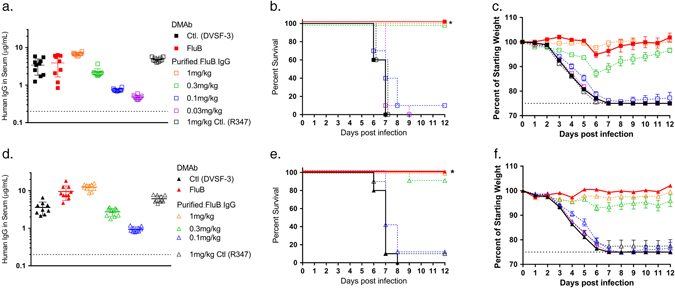
FluB DMAb protects mice from diverse lethal influenza B challenges. BALB/c mice were treated with FluB DMAb plasmid DNA 5 days prior to infection with B/Malaysia/2506/2004 Victoria (**a–c**) or B/Florida/4/2006 Yamagata (**d–f**) lineage virus. One day prior to infection, separate groups of mice received 0.03–1 mg/kg FluB protein monoclonal antibody i.p. **a**, **d** Human IgG in mouse sera at the time of infection. *Dotted line* indicates LOD (*n* = 10 animals per group, mean ± SD). **b**, **e** Kaplan–Meier survival curves of BALB/c mice challenged with influenza B (*n* = 10 animals per group, *p ≤ 0.0001 FluB DMAb versus Control DMAb). **c**, **f** Weight of BALB/c mice following influenza B challenge. *Dotted line* indicates 25% maximum weight loss. (*n* = 10 animals per group, mean ± SEM)

To evaluate the protective capability of the FluB DMAb, we performed similar lethal challenge studies with influenza B. In these studies, mice were administered 200 μg FluB DMAb construct or control DMAb construct via IM-EP, then challenged with a lethal dose of virus from the Victoria (B/Malaysia/2506/2004 (B/Mal/04)) or Yamagata lineage (B/Florida/4/2006 (B/Fla/06)) 5 days later (Fig. 4). Again, for direct comparison of DMAb versus purified protein, recombinant FluB monoclonal antibody was administered i.p. to separate groups 1 day prior to infection. Quantification of human IgG present in mouse serum at time of challenge showed that FluB DMAb yielded similar mean human IgG concentrations and HA binding activity as observed in animals treated with 1 mg/kg of FluB protein i.p. (Fig. 4a, d, Supplemental Fig. S3). Remarkably, 100% of FluB DMAb-treated mice survived both Victoria and Yamagata lethal influenza B challenge, whereas non-specific DMAb controls fully succumbed to both infections by Day 8 (Fig. 4b, e). Consistent with survival data, FluB DMAb protected mice from influenza B-related morbidity with treated animals exhibiting little-to-no weight loss (Fig. 4c, f). In addition, FluB DMAb-treated mice exhibited significantly lower lung viral loads than those observed in control mice (Supplemental Fig. S4). Survival, weight loss, lung viral loads, and in vitro binding activity in sera of FluB DMAb-treated mice closely paralleled the same parameters in mice receiving 1 mg/kg purified FluB protein IgG, again confirming the in vivo functional equivalence of DMAb and purified recombinant monoclonal antibodies.

### Co-administration of FluA and FluB DMAb protects mice against influenza A and B challenge, and homologous re-challenge

**Fig. 5:**
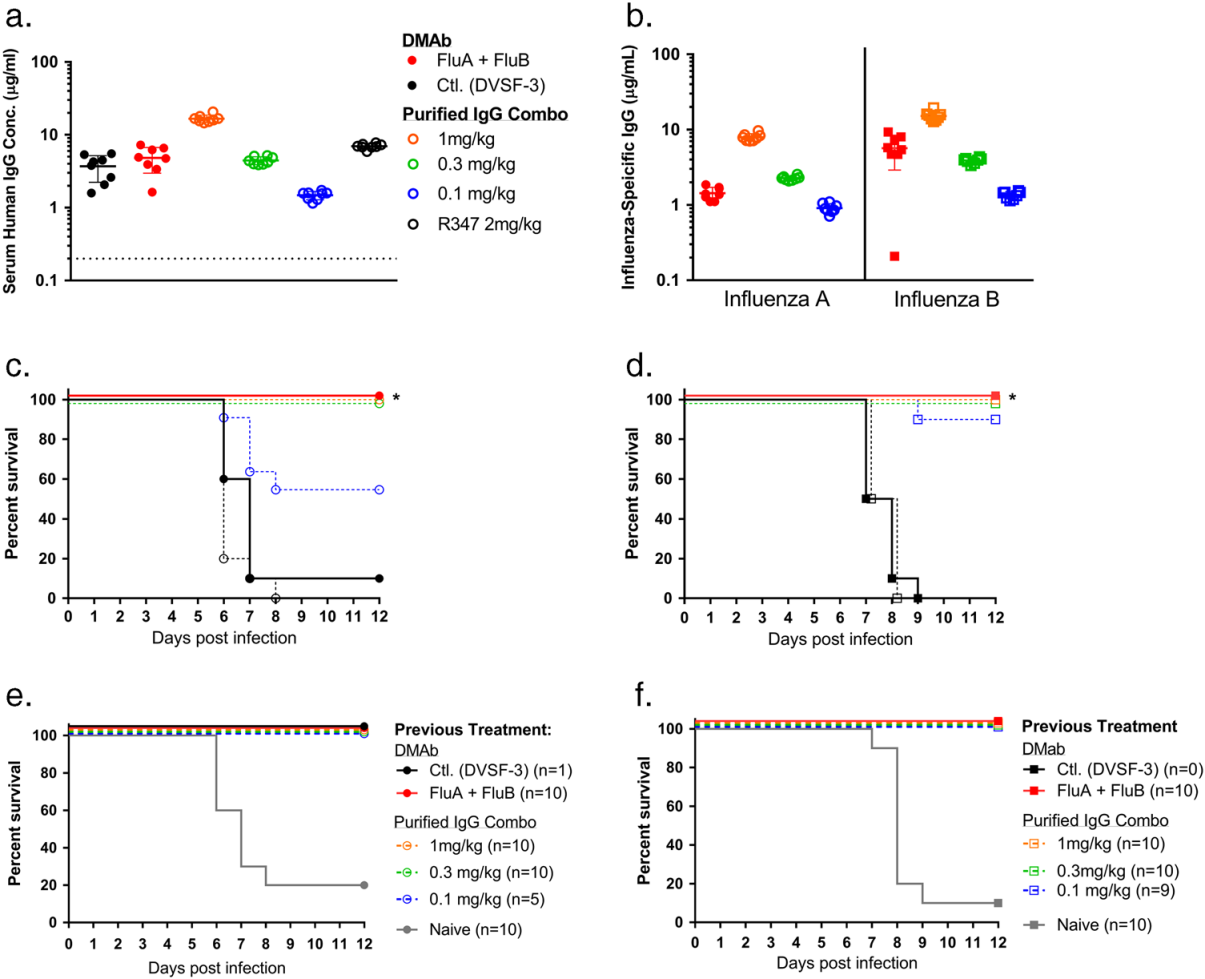
Co-administration of FluA and FluB DMAb protects mice from lethal influenza A/B challenge and homologous re-challenge. BALB/c mice received both FluA and FluB DMAb. Separate mice were treated with both FluA plus FluB protein monoclonal antibody. Mice received initial infection with either influenza A/California/7/2009 or B/Florida/4/2006. **a** Total human IgG levels in mice sera at the time of infection (*n* = 8 animals per group, mean ± SD). **b** Influenza A-specific and B-specific human IgG in mouse serum at the time of infection quantified by HA binding ELISA (*n* = 8 animals per group, mean ± SD). **c**, **d** Kaplan–Meier survival curves following initial infection with A/California/07/2009 (**c**) or B/Florida/4/2006 (**d**) (*n* = 10 animals per group, *p ≤ 0.0001 Flu DMAb versus control DMAb). **e**, **f** 28 days following initial infection, surviving mice received homologous influenza re-infection. Kaplan–Meier survival curves following re-infection, compared to mice receiving neither DMAb/IgG treatment nor initial infection (naïve). (Number of animals in each group are shown)

Influenza A and B viruses co-circulate, and a comprehensive immunoprophylactic strategy against seasonal infection should target both influenza types. To test the ability of the DMAb platform to serve in this role, both FluA DMAb and FluB DMAb constructs were administered to BALB/c mice. Comparator groups of animals received a mix of purified recombinant FluA and FluB monoclonal antibodies i.p. Mice were challenged with a lethal dose of either A/CA/09 H1 or B/Fla/06. Serum samples at the time of infection showed that the DMAb-treated animals had an average of 3 μg/mL of total human IgG (Fig. 5a). Influenza A-specific and B-specific ELISAs showed that FluA and FluB DMAbs exhibited expression levels similar to those observed in sera of mice receiving 0.3 mg/kg of recombinant FluA monoclonal antibody and 1 mg/kg recombinant FluB monoclonal antibody delivered i.p., respectively (Fig. 5b). All mice receiving FluA plus FluB DMAb were completely protected from lethal infection, whereas 90% and 100% of mice treated with control DMAb succumbed to the influenza A and B infections, respectively (Fig. 5c, d). DMAb administration and delivery of protein IgG resulted in similar levels of protection, apparent in both survival rate and body weight loss (Supplemental Fig. S5).

Twenty-one days following initial infection, sera of surviving BALB/c mice had undetectable levels of human IgG (data not shown), indicating DMAb and recombinant protein were no longer present. Serum hemagglutination inhibition (HAI) and mouse anti-HA binding antibodies against the infecting influenza strain confirmed that mice mounted a host immune response to infection (Supplemental Fig. S6). DMAb-treated mice were able to mount host immune responses against the virus to the same extent as the purified IgG-treated animals.

Twenty-eight days following initial infection, all surviving mice (including one DMAb control mouse that survived initial A/CA/09 H1 infection) were re-challenged with a lethal dose of homologous influenza virus to confirm that the level of mouse host immune response was protective. All previously-challenged mice survived the lethal homologous re-challenge without substantial weight loss, whereas 80–90% of untreated age-matched mice naïve to infection did not survive (Fig. 5e, f, Supplemental Fig. S5). These results demonstrate protective host anti-influenza responses developed in the presence of protective levels of FluA and FluB antibodies whether expressed in vivo as DMAb or delivered as protein monoclonal antibody, demonstrating that DMAbs did not antagonize each other or the host immune response to influenza.

## Discussion

Seasonal influenza infection results in an annual average of $10 billion USD in direct medical costs and $80 billion USD economic burden in the United States alone.^18^ Despite availability of influenza vaccines and anti-viral drugs, large sub-populations remain susceptible to complications arising from seasonal influenza infection. Almost 90% of deaths attributed to seasonal influenza in the United States occur in adults 65 years and older,^19^ a population in which estimated vaccine efficacy is as low as 36% in years of significant antigenic drift (http://www.cdc.gov/flu/professionals/vaccination/effectivenessqa.htm) with standard vaccine technology. In addition to the persistent hazards of seasonal infection, pandemic influenza outbreaks threaten to outpace vaccine design. Therefore, innovative universal interventions against influenza infection are vital.

Most of the current efforts to create a universal influenza vaccine have focused on the design of recombinant antigens that can serve as immunogens to spur active immunity and maturation of host cross-protective anti-influenza antibodies.^20–22^ The concordant discovery of novel broadly-neutralizing anti-influenza monoclonal antibodies suggests passive immunization may complement or bypass traditional immunization strategies against influenza. Here, we sought to bypass active immunization and generate cross-protective antibody responses via inoculation with DMAbs. We show that this approach generated functional anti-influenza antibodies in mouse sera following inoculation with plasmid DNA constructs encoding two antibodies against HA from influenza subtypes known to cause disease in humans, leading to significant protection against lethal seasonal influenza A and influenza B challenges. Studies are underway to further enhance DMAb expression, and to assess the in vivo capability of DMAb to protect against highly pathogenic avian influenza viruses and other potentially pandemic strains.

A plethora of protein monoclonal antibodies are commercially available for treatment of auto-immune disease, cancer, and other chronic conditions. Yet given the expense of administering biologics, and their limited half-life, only one protein monoclonal antibody has been approved and is routinely used for prophylaxis against an infectious disease target (palivizumab, RSV).^23^ DMAb technology is a notable delivery alternative as DMAb produced from muscle cells in vivo and purified monoclonal antibodies manufactured in vitro confer similar levels of protection against lethal influenza infection in mice. Plasmid DNA lacks limitations posed by pre-existing anti-vector serology and the DMAb platform may be utilized repeatedly to deliver additional anti-influenza antibodies to combat viral escape, or antibodies aimed at entirely different pathogens.^16, 17^ Plasmid DNA also has little risk of genomic integration (reviewed in ref. 24) and similar plasmid designs have demonstrated safety in DNA vaccine human clinical studies.^25^

DNA plasmid-based delivery of monoclonal antibodies provides conceptual advantages at each step of the supply chain. In production, DMAb are inexpensive relative to protein monoclonal antibody (and viral vectors) because DNA replication does not require mammalian cell culture. DNA is simple to scale up and stable for storage, a particularly important consideration in resource-limited settings. The potential for long-term DMAb expression with sustainable passive immune protection against influenza infection may circumvent the need for frequent recombinant antibody injections. Long-term expression of DMAb can be further augmented by emerging antibody half-life extension technologies.^26^ Conceivably, inoculation with influenza-specific DMAbs may have utility to augment a vaccine campaign, generating almost immediate prophylaxis against severe influenza infection while allowing for an adequate vaccine-induced immune response to mature. DMAbs may also provide a vital option for severely immune impaired individuals incapable of mounting functional active immune responses. DMAb technology is an exceptionally compelling tool warranting further exploration for sustained protection against severe infection by diverse strains influenza A and B viruses, as well as further investigation against infectious diseases more generally.

## Materials and methods

### DNA-encoded monoclonal antibody (DMAb) constructs

Monoclonal antibodies were isolated using similar methodology as described previously.^10, 14, 15^ The influenza A monoclonal antibody (FluA) was isolated based on cross-reactive binding to H5 and H7 HA proteins^10^ and the influenza B monoclonal antibody (FluB) was isolated based on neutralization activity against distinct lineages of influenza B. Variable gene sequences were isolated from cross-reactive clones by reverse transcription polymerase chain reaction, cloned, and further modified to revert nonessential non-germline framework amino acid changes. Full-length human IgG1κ were transiently expressed in CHO cells and purified for use in in vivo studies. Plasmid DMAb constructs were engineered as previously described.^16, 17^ DMAb constructs encoded fully human IgG1κ monoclonal antibodies FluA DMAb and FluB DMAb. Antibody amino acid sequences were DNA codon-optimized and RNA-optimized for expression in human/mouse, and resulting DNA transgenes were synthesized de novo (Genscript, Piscataway, NJ, USA). These synthetic transgenes were restriction-cloned into a modified pVax1 mammalian expression vector (Invitrogen) under the cytomegalovirus immediate-early promoter. IgE heavy-chain and light-chain leader sequences were added for cellular processing and secretion. In initial studies (Figs. 1–4), transgenes consisted of antibody heavy-chain and light-chain sequences separated by a furin/picornavirus-2A peptide cleavage site sequence, yielding expression of heavy-chain and light-chain peptides from a single plasmid. In later studies with co-administration of FluA and FluB DMAb (Fig. 5), two FluA DMAb constructs individually expressing heavy-chain or light-chain FluA peptides were mixed for expression of heavy-chain and light-chain FluA peptides from separate plasmids.

### Transfection and western blot

Human 293 T cells (ATCC) were maintained in Dulbeco’s modified Eagle medium (Invitrogen) supplemented with 10% fetal bovine serum (FBS). One day prior to transfection, cells were plated at 0.25 × 10^6^ cells per well in a 12-well plate and transfected with 0.5 μg plasmid DNA using GeneJammer (Agilent Technologies). 48 h later, supernatants were collected and adherent cells were lysed with 1× cell lysis buffer (Cell Signaling) with protease inhibitor cocktail (Roche Boehringer Mannheim). Approximately 50 μg of total supernatant/lysate protein and 10 μg of protein IgG were run with SeeBlue Plus2 pre-stained protein standard (Thermo Fisher Scientific) on precast 4–12% bis-tris gels (Invitrogen) and transferred to an Immobilon-FL PVDF membrane (EMD Millipore) using the iBlot 2 Dry Blotting System (Thermo Fisher Scientific). Heavy-chain and light-chain peptides were identified using IRDye 800CW goat anti-human IgG (H + L) (LI-COR Biosciences) (1:10,000). Fluorescent blots were scanned with the Odyssey CLx (LI-COR Biosciences).

### Quantitative ELISA

For quantification of total human IgG1κ in cell lysates, cell supernatants, and mouse sera in Fig. 1 and Supplemental Fig. S1, 96-well MaxiSorp plates (Nunc) were coated overnight at 4 °C with 10 μg/mL goat anti-human IgG F_c_ fragment (Bethyl Laboratories). Plates were blocked with 10% FBS in phosphate-buffered saline (PBS). Sample was diluted in 1× PBS + 0.1% Tween_20_ and added to plates for 1 h. A standard curve was generated using purified human IgG1κ (Bethyl Laboratories). Plates were stained with HRP-conjugated secondary antibody goat anti-human kappa light-chain (Bethyl Laboratories) (1:20,000) for 1 h and developed using SigmaFast OPD (Sigma-Aldrich). Absorbance at an OD of 450 nm was measured on a Synergy2 plate reader (Biotek).

Quantitation of human IgG in murine challenge studies was performed using 384-well black MaxiSorp plates (Nalgene Nunc) coated overnight at 4 °C with 10 μg/mL goat anti-Human IgG (H + L) (Pierce). Plates were blocked with Casein Blocker (Thermo), and serum samples and a standard curve (10 μg/mL of ChromPure Human IgG, whole molecule) (Jackson Labs) were serially diluted. Plates were washed and stained with a donkey anti-Human IgG-HRP secondary antibody (Jackson) (1:4,000) and visualized using SuperSignal ELISA Pico Reagent (Thermo). Luminescence was measured using Perkin Elmer Envision.

Quantification of specific influenza A or B human IgG in the sera of mice was performed as described above, with 3 μg/mL of HA protein from A/Vietnam/1203/2004 (H5N1) or 3 μg/mL of HA from B/Florida/4/2006 (Yamagata) as coating reagent. FluA or FluB purified protein IgG were used as standards for the influenza A and B assays, respectively.

### Binding ELISA

Recombinant HA proteins were expressed and purified as previously described.^27^ ELISA binding assays were performed using 384-well MaxiSorp plates (Nunc) coated with 5 µg/mL of purified HA protein from A/Perth/16/2009 (H3N2), A/Hong Kong/G9/1997 (H9N2), and B/Brisbane/60/2008 (Victoria); or 3 µg/mL of purified HA protein from A/California/07/2009 (H1N1), A/Vietnam/1203/2004 (H5N1), A/Netherlands/2003 (H7N7), A/Missouri/2006 (H2N3), and B/Florida/4/2006 (Yamagata). ELISA plates were blocked with Casein (Thermo Scientific) and serially diluted antibodies were incubated for 1 h at room temperature. Bound antibodies were detected using a peroxidase-conjugated mouse anti-human IgG antibody (KPL) (1:10,000), followed by development with TMB solution (KPL), and absorbance measurement at an OD of 450 nm. Mouse serum reactivity to HA was preformed as described above with the exception of secondary antibody of peroxidase-conjugated goat anti-mouse IgG antibody (DAKO) (1:5,000).

### Viral stocks, neutralization, and hemmaglutination inhibition

Wild-type influenza strains were obtained from the Centers for Disease Control and Prevention, or purchased from the American Tissue Culture Collection. A re-assortant H3 virus produced by reverse genetics (rA/HK/68) contained the H3 HA from A/Hong Kong/8/68 (H3N2) and the remaining seven gene segments from A/Puerto Rico/8/34 (H1N1); the HA of this virus also contained a N165S mutation that enhances murine pathogenesis.^28^ All viruses were propagated in embryonated chicken eggs, and virus titers were determined by mean 50% tissue culture infective dose (TCID_50_) per milliliter. The microneutralization assay was performed as previously described.^27^ Briefly, 60 TCID_50_ of virus per well was added to three-fold serial dilutions of serum or purified FluB antibody diluted in naïve serum in a 384-well plate in complete MEM medium containing 0.75 μg/mL N-tosyl-L-phenylalanyl chloromethyl keytone Trypsin (Worthington) in duplicate wells. After 1-h incubation at 33 °C and 5% CO_2_, 2 × 10^4^ Madin–Darby Canine Kidney cells per well were added to the plate. Plates were incubated at 33 °C and 5% CO_2_ for approximately 40 h, and NA activity was measured by adding a fluorescently-labeled substrate methylumbelliferyl-N-acetyl neuraminic acid (MU-NANA) (Sigma) to each well at 37 °C for 1 h. Virus replication represented by NA activity was quantified by reading fluorescence using the following settings: excitation 355 nm, emission 460 nm, ten flashes per well. HAI assay was performed with serum collected on Day 21 post infection as previously described.^27^

### Intramuscular DNA electroporation

Thirty minutes prior to DNA electroporation, female BALB/c and CAnN.Cg-*Foxn1* ^*nu*^/Crl mice (Charles River) were pre-treated at each delivery site with an intramuscular (i.m.) injection of 12 Units (30 μL) hyaluronidase enzyme (Sigma-Aldrich). Treatment groups were selected at random and data collection was not blinded. In initial studies (Figs. 1–4), 100 μg (30 μL) of either FluA or FluB DMAb plasmid was injected i.m. to the tibialis anterior (TA) and/or quadriceps (Q) muscle; mice received 100 μg DNA at one site (TA), 200 μg DNA at two sites (right TA + left TA), or 300 μg DNA at three sites (right TA + left TA + Q). In later co-administration studies (Fig. 5), mice received both FluA and FluB DMAb constructs. We modified the FluA construct design to express heavy-chain and light-chain peptides on separate plasmids, generating equivalent serum levels of FluA IgG from fewer injection sites than the one-plasmid design. One hundred μg of a 1:1 (wt:wt) mixture of FluA heavy-chain and light-chain plasmid were delivered over two sites (right TA + right Q), and 200-μg plasmid FluB was delivered over two sites as before (left TA + left Q). IM-EP was performed immediately after each DNA injection with a CELLECTRA® 3P adaptive constant current device (Inovio Pharmaceuticals).^13, 29, 30^

### Lethal influenza challenge

Six- to eight-week-old BALB/c mice (Harlan Laboratories) received FluA DMAb, FluB DMAb, or an irrelevant control DMAb (DVSF-3, previously described)^17^ via IM-EP 4–5 days prior to infection. One day prior to infection, protein IgG monoclonal antibody with amino acid sequence identical to that encoded by plasmid DMAb was administered to separate groups of mice i.p. at doses ranging from 0.03 to 1.0 mg/kg. Control mice received non-specific protein IgG R347 i.p. Mice received intranasal infection with 3 × LD_50_ of A/California/07/2009 (H1N1) (9.5 × 10^4^ TCID_50_/mouse), 7 × LD_50_ of rA/HK/68 (H3) (1.2 × 10^5^ TCID_50_/mouse), 10 × LD_50_ B/Malaysia/2506/2004 (Victoria) (3.6 × 10^4^ TCID_50_/mouse), or 7 × LD_50_ B/Florida/4/2006 (Yamagata) (7.0 × 10^4^ TCID_50_/mouse). All mice were monitored daily for weight loss and survival for 12 days. The percent change in weight was calculated based on the pre-infection weight. Animals that lost ≥ 25% of their total weight were euthanized, and weight loss was recorded as the limit for the remainder of the study. Blood was collected on the day of infection to assess the amount of human IgG in the serum. To assess viral load in the lungs, additional mice were euthanized 5 days post infection. In homologous re-infection studies, blood samples were taken from all surviving mice 21 days after initial infection to confirm clearance and absence of human IgG (data not shown). Twenty-eight days after the initial infection, previously-infected surviving mice were re-challenged with a virus strain and lethal dose identical to the initial infection (alongside age-matched naïve controls).

All animal housing and experimentation were approved by and conducted in accordance with the guidelines set by the National Institutes of Health, the Animal Care and Use Review Office of the U.S. Army Medical Department, the University of Pennsylvania Perelman School of Medicine Institutional Animal Care and Use Committee, and MedImmune Institutional Animal Care and Use Committee. All murine challenge studies were conducted in accordance with and subsequently performed in Association for the Assessment and Accreditation of Laboratory Animal Care-certified facilities.

### Analyses and statistics

Standard curves and graphs were prepared using GraphPad Prism 6. EC_50_ and IC_50_ values were calculated using a non-linear regression of log (reciprocal serum dilution) vs response. Survival data were expressed using Kaplan–Meier survival curves with *p*-values calculated by log-rank (Mantel–Cox) test.

### Data availability

Data that support the findings of this study are available from the corresponding author upon reasonable request.

## Electronic supplementary material

Supplemental Figure S6

Supplemental Figure S5

Supplemental Materials

Supplemental Figure S1

Supplemental Figure S2

Supplemental Figure S3

Supplemental Figure S4
