# Supplementary figures and images for: DMAb inoculation of synthetic cross reactive antibodies protects against lethal influenza A and B infections
Source: NPJ Vaccines. 2017 Jul 6;2:18. doi: 10.1038/s41541-017-0020-x (PMC5627301; doi:10.1038/s41541-017-0020-x)

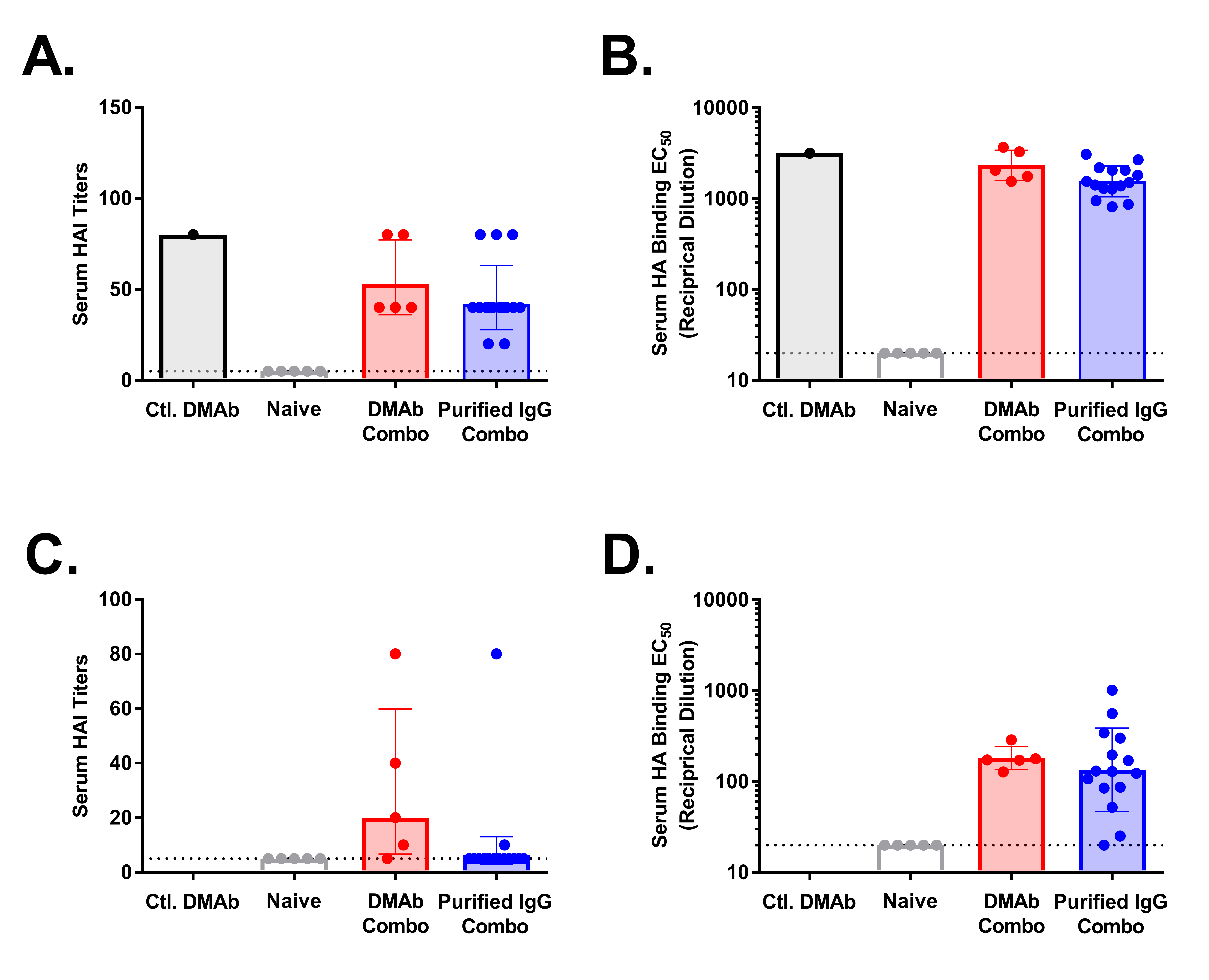

Supplement: Supplementary file 1 — Supplemental Figure S6 [file 41541_2017_20_MOESM1_ESM.tif]

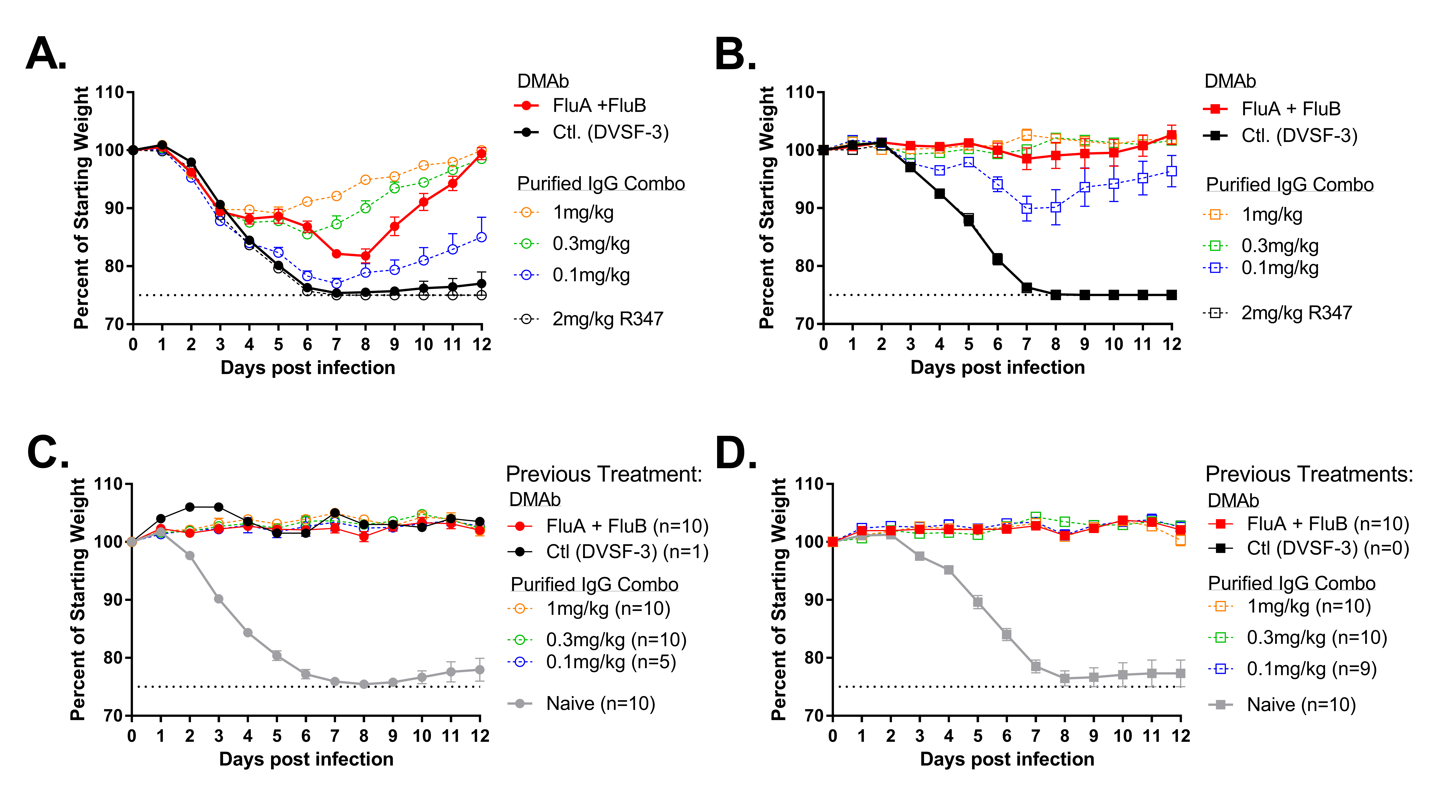

Supplement: Supplementary file 2 — Supplemental Figure S5 [file 41541_2017_20_MOESM2_ESM.tif]

## Slide 1
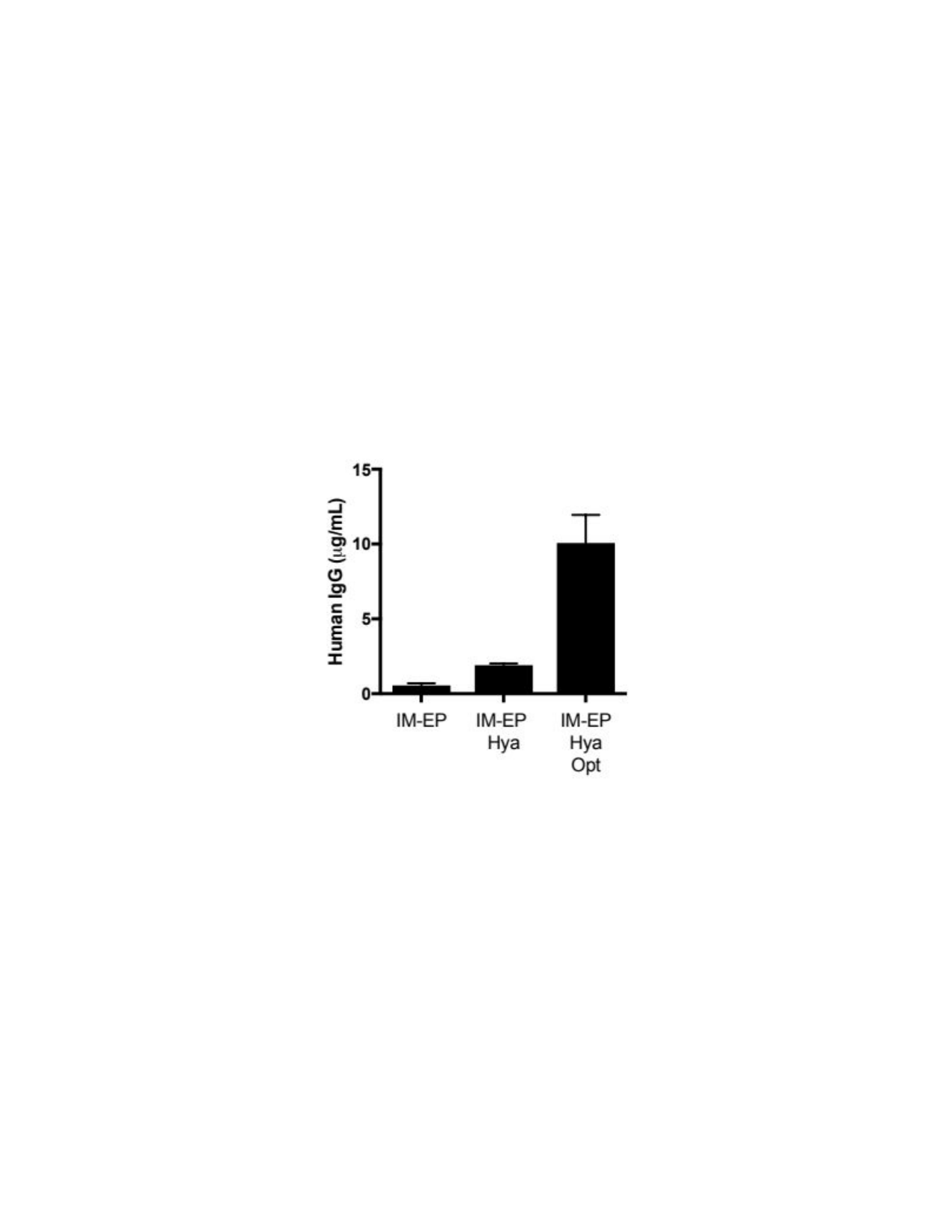

Supplement: Supplementary file 4 — Supplemental Figure S1 [file 41541_2017_20_MOESM4_ESM.pptx]

## Slide 1
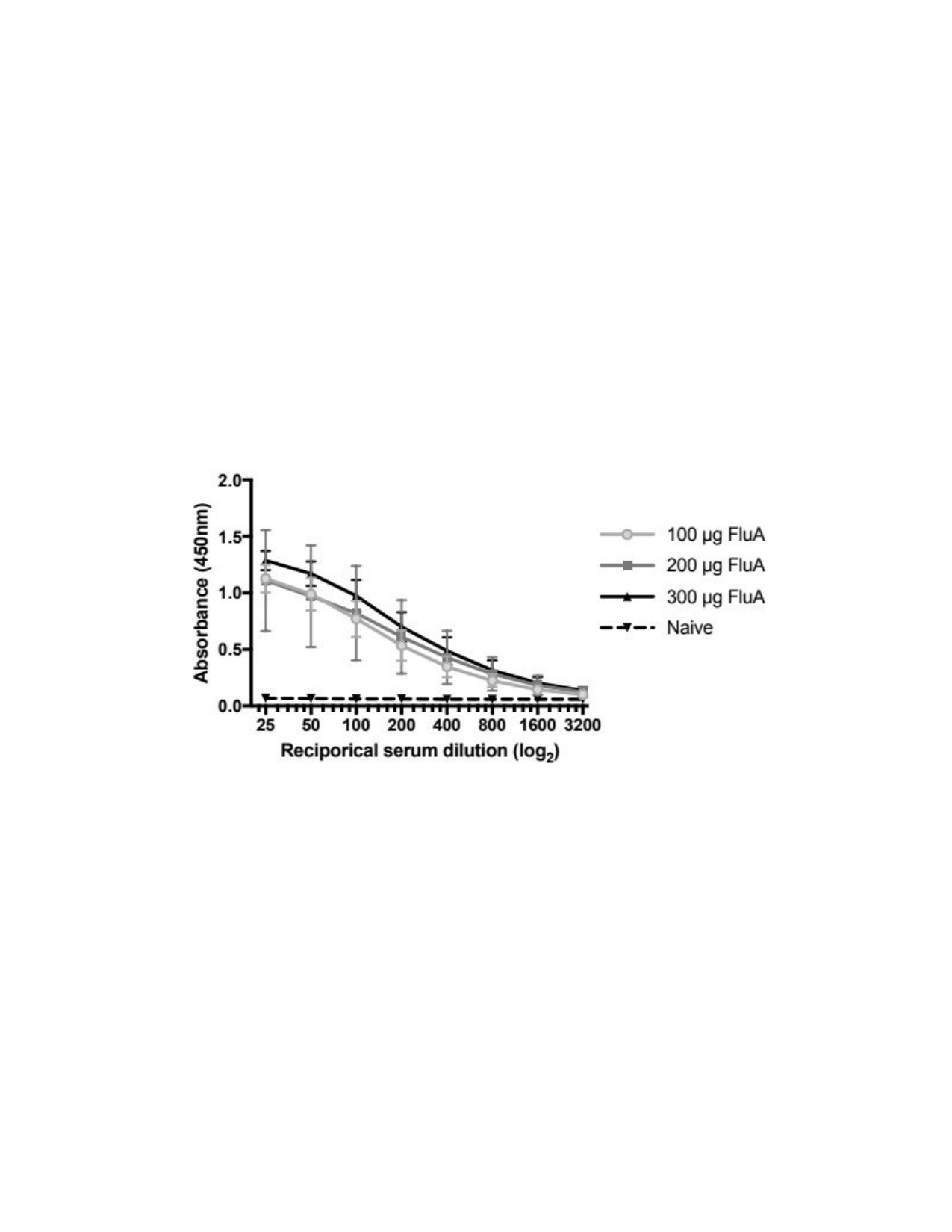

Supplement: Supplementary file 5 — Supplemental Figure S2 [file 41541_2017_20_MOESM5_ESM.pptx]

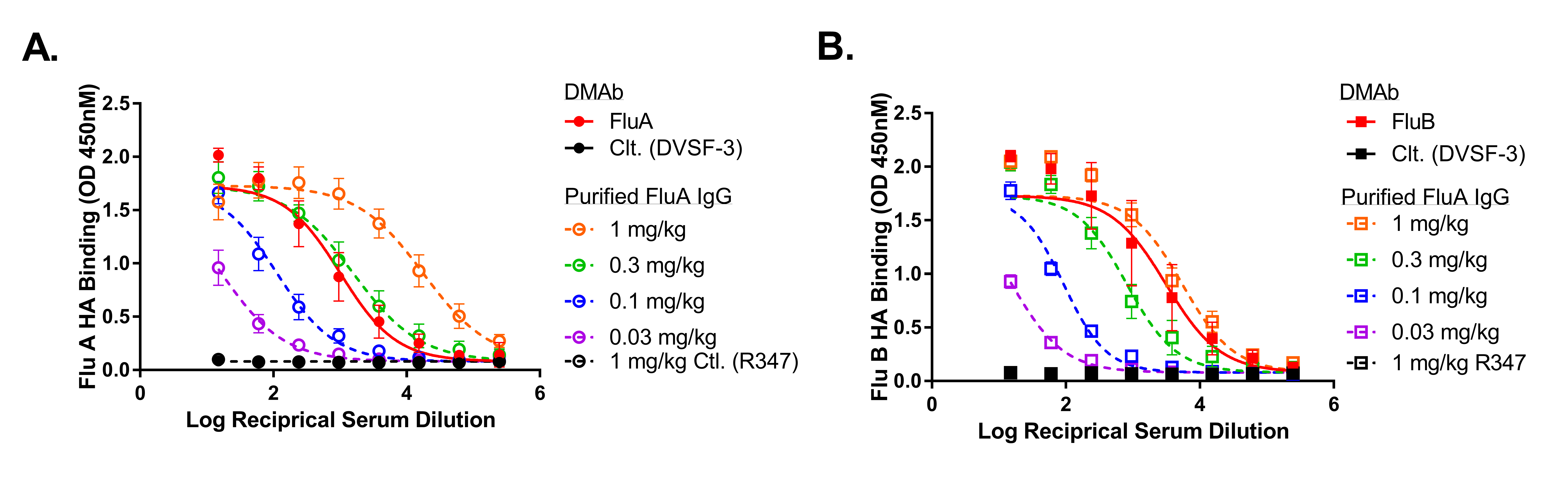

Supplement: Supplementary file 6 — Supplemental Figure S3 [file 41541_2017_20_MOESM6_ESM.tif]

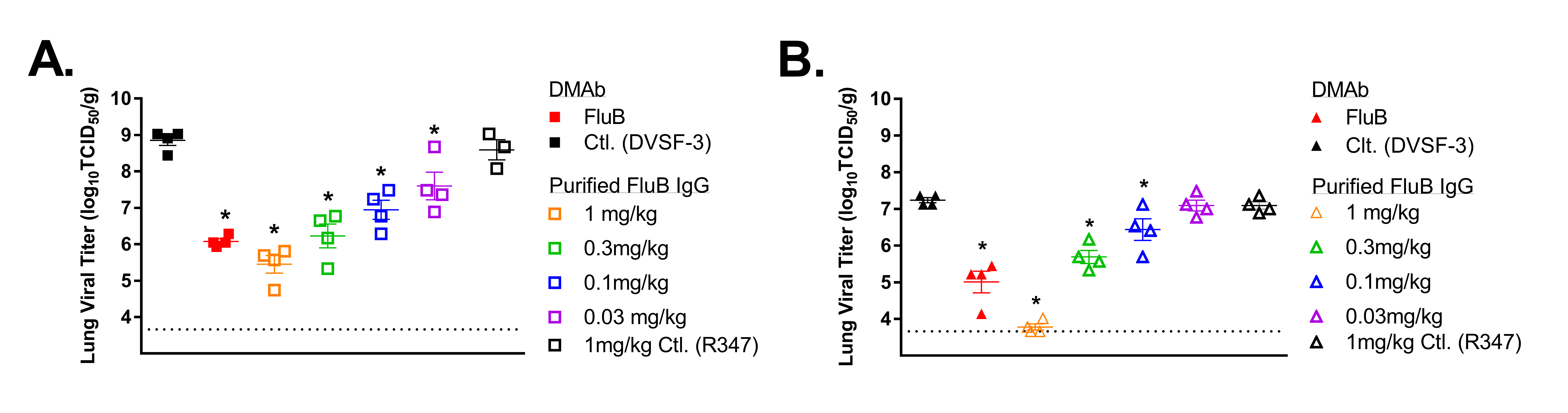

Supplement: Supplementary file 7 — Supplemental Figure S4 [file 41541_2017_20_MOESM7_ESM.tif]
