# Supplementary material for: DMAb inoculation of synthetic cross reactive antibodies protects against lethal influenza A and B infections
Source: NPJ Vaccines. 2017 Jul 6;2:18. doi: 10.1038/s41541-017-0020-x (PMC5627301; doi:10.1038/s41541-017-0020-x)
Supplement: Supplementary file 3 — Supplemental Materials [file 41541_2017_20_MOESM3_ESM.pdf]

Supplementary Materials:

Supplemental Figure S1: Modifications enhance *in vivo* DMAb expression.

Supplemental Figure S2: FluA DMAb in sera binds influenza A hemagglutinin H10.

Supplemental Figure S3: Protein IgG and FluA/FluB DMAb expressed *in vivo* exhibit similar hemagglutinin binding.

Supplemental Figure S4: FluB significantly lowers influenza B viral burden in lungs.

Supplemental Figure S5: Co-administration of FluA and FluB DMAb protects mice from lethal influenza challenge and homologous re-challenge.

Supplemental Figure S6: Serum reactivity of DMAb-treated mice 21 days post-infection.

Supplemental Figure S1: Modifications enhance *in vivo* DMAb expression. Serum DMAb human IgG expression in mice five days following sequentially modified administrations of 200 μg FluB plasmid DNA. Plasmid DNA was delivered to BALB/C mice via intramuscular electroporation alone (IM-EP), or via IM-EP with hyaluronidase formulation (Hya + IM-EP). Furthermore, plasmid transgene insert sequences were DNA codon-optimized and RNA-optimized for enhanced expression (Opt + Hya + IM-EP). There was no detectable DMAb expression in the absence of electroporation. All other in vivo studies were performed Opt + Hya + IM-EP. (n=5 animals per group, mean ±SEM).

Supplemental Figure S2: FluA DMAb in mouse sera binds influenza A hemagglutinin H10. Sera from BALB/c mice collected 5 days after treatment with 100-300 μg of FluA DMAb plasmid DNA were serially diluted and added to 96-well plates coated with influenza A Group 2 recombinant H10 antigen (A/Jiangxi-Donghu/346/2013 H10N8) (IBT Bioservices). DMAb binding was detected with HRP-conjugated secondary antibody donkey anti-human IgG (1:5,000) and developed using SigmaFast OPD substrate (Sigma-Aldrich). Absorbance was measured at 450 nm. Sera from un-treated (naïve) mice served as a control. (n=5 animals per group, mean ±SD).

Supplemental Figure S3: Protein IgG and FluA/FluB DMAb expressed *in vivo* exhibit similar hemagglutinin binding. Serum samples from BALB/C mice treated with FluA (A) or FluB (B) DMAb plasmid DNA, irrelevant control DMAb construct (DVSF-3), purified anti-influenza IgG protein, or irrelevant control IgG were harvested on the day of influenza infection and tested for HA reactivity by binding ELISA. **(A)** Reactivity to purified H1 HA protein from A/California/7/2009 H1 or **(B)** reactivity to purified Victoria lineage HA protein from B/Brisbane60/2008 Victoria. (n=10 animals per group, mean ±SD).

Supplemental Figure S4: FluB significantly lowers influenza B viral burden in lungs. BALB/c mice were treated with 200 μg FluB DMAb plasmid DNA or irrelevant DMAb control construct (DVSF-3) 5 days prior to infection. Separate groups received 0.03-1 mg/kg FluB purified IgG protein or irrelevant control IgG R347 i.p. one day prior to infection. Whole lungs were homogenized in 10% (wt/vol) sterile L15 medium (Invitrogen) and titrated on MDCK cells to determine the $TCID_{50}$/gram of tissue. Lung Viral Titers on day 5 post-infection with **(A)** B/Malaysia/2508/2004 or **(B)** B/Florida/4/2006. (n=4 animals per group, mean ±SEM). Dotted line indicates LOD. * Significant reduction in viral titers compared to control DMAb DVSF-3 group by Student's t test.

Supplemental Figure S5: Co-administration of FluA and FluB DMAb protects mice from lethal influenza challenge and homologous re-challenge. BALB/C mice received both FluA and FluB DMAb constructs. Separate groups were treated with 0.1-1 mg/kg of a combination of FluA and FluB protein IgG one day prior to infection. Body weight loss of animals infected with **(A)** A/California/7/2009 (n=10 animals per group, mean ±SEM) or **(B)** B/Florida/4/2006 (n=10 animals per group, mean ±SEM). **(C,D)** Body weight loss following homologous influenza re-challenge of surviving mice with A/California/7/2009 (C) or B/Florida/4/2006 (D) 28 days following initial infection. (Number in each group indicated, mean ±SEM). Dotted line indicates 25% maximum weight loss.

Supplemental Figure S6: Serum reactivity of DMAb-treated mice 21 days post-infection. Functional assays performed with sera from surviving BALB/c mice collected 21 days after infection with A/California/7/2009 (A, B) or B/Florida/4/2006 (C, D). **(A)** Hemagglutination inhibition activity (reciprocal dilution) against infecting virus A/California/07/2009. **(B)** ELISA binding EC_{50} values (reciprocal dilution) to influenza A/California/07/2009 HA protein. **(C)**

Hemagglutination inhibition activity (reciprocal dilution) against infecting virus B/Florida/4/2006. **(D)** ELISA Binding EC_{50} values (reciprocal dilution) to influenza B HA protein. (Number of mice in each group indicated by number of symbols, mean ±SD).
